# Current challenges of high-solid anaerobic digestion and possible measures for its effective applications: a review

**DOI:** 10.1186/s13068-022-02151-9

**Published:** 2022-05-18

**Authors:** Julius G. Akinbomi, Regina J. Patinvoh, Mohammad J. Taherzadeh

**Affiliations:** 1 Department of Chemical Engineering, Faculty of Engineering, Lagos State University, Lagos, 100268 Nigeria; 2 Swedish Centre for Resource Recovery, University of Borås, 50190 Borås, Sweden

**Keywords:** Waste management, Anaerobic digestion, High solids, Challenges, Inhibition, Improved measures

## Abstract

The attention that high solids anaerobic digestion process (HS-AD) has received over the years, as a waste management and energy recovery process when compared to low solids anaerobic digestion process, can be attributed to its associated benefits including water conservation and smaller digester foot print. However, high solid content of the feedstock involved in the digestion process poses a barrier to the process stability and performance if it is not well managed. In this review, various limitations to effective performance of the HS-AD process, as well as, the possible measures highlighted in various research studies were garnered to serve as a guide for effective industrial application of this technology. A proposed design concept for overcoming substrate and product inhibition thereby improving methane yield and process stability was recommended for optimum performance of the HS-AD process.

## Introduction

Given the mounting and daunting climate change and global warming situation, it is now inevitable for humankind to adopt a paradigm shift from conventional and non-environmentally friendly approach of waste disposal and fossil fuels energy consumption, to more environmentally benign approaches and circular bioeconomy. It is evident that the world may not be able to survive the catastrophic consequences of unchecked climate change [1, 2]. Indiscriminate dumping and landfilling of organic wastes are common traditional linear economy being practiced in most countries, However, a sustainable world demand a circular behaviour and converting these wastes back to resources [3]. One of the environmentally friendly approaches for biodegradable waste disposal and energy recovery that has been adopted, though not optimally, is anaerobic digestion (AD) [4]. Green energy and value added products, including organic fertilizers are produced from anaerobic digestion of biodegradable wastes [5–7]. AD is a process through which biodegradable organic wastes are broken down in the absence of air by microbial activities with consequent production of biogas and digestates that could be used for energy and soil fertilization purposes, respectively. It is a slow process with a typical retention time in the bioreactors of about 20–40 days [8].

Total solids (TS) is one of the important factors of AD processes, which can be classified into two main categories based on the total solid (TS) content of the solid wastes; low solid or wet anaerobic digestion (LS-AD) and high solid or dry anaerobic digestion (HS-AD). LS-AD and HS-AD processes involves the decomposition of biodegradable wastes with TS content ≤ 10% and ≥ 20% respectively [9]. Major comparison on the LS-AD and HS-AD are shown in Table 1. The form of anaerobic digestion process that is commonly practiced all over the world is LS-AD because of the simplicity of the technology involved [10, 11]. However, LS-AD has some hitches that affect the optimal productivity of the system. One of the constraints include the requirement of large digester footprint due to low TS content of the feedstock; the large volume digester is needed to provide for optimal valorisation of the feedstock. Another constraint of the LS-AD is the demand for large quantity of water to reduce the solid content of the feedstock, which is a major challenge for the regions with shortage of water [12–14]. This requirement places huge pressure on water resource which becomes a challenge as population increases and industrial development progresses. Beside, the high amount of water used during LS-AD often results in production of digestate with large volume of water that will, in turn, require use of screw press or chemical separation to reduce water level of the digestate before further application for agricultural use. Other challenges related to the application of LS-AD include cost implication and energy requirement of equipment such as pumps, mixers, thickeners, among others.

**Table 1:** Comparative study on LS-AD and HS-AD plants in UK and Europe [3]

Parameters	Wet anaerobic digestion process	High solids anaerobic digestion process	Remarks
Feedstock solids content	The total solids content was lower but the volatile solids content was higher compared to high solids AD plants	The total solids content was higher but the volatile solids content was lower compared to wet AD plants	The feedstock TS ranged from 17 to 50% and the volatile solids (VS), as a percentage of the TS, was ranged from 50 to 96%
Water usage	Water usage was higher	Water usage was lower	The high solids content of feedstock for high solids AD plants decreased process water consumption
Plant footprint relative to plant design capacity	Total plant footprint of wet AD plant was influenced by the digestate storage facility	Total plant footprint of high solids AD plants was determined by the pre-and post-digestion treatments	Total plant footprints varied from 2900 to 80,000m^2^ while the design waste processing capacities varied from 27, 500 to 287, 500 tons per year. There was, however, no significant difference of the plant footprints relative to the plant design capacity between the wet and high solids AD plants
Pre-treatment and post- treatment facilities	Wet AD plants in most cases do not have complex pre-treatment and post- treatment facilities but instead have storage facilities	High solids AD plants have more complex pre-treatment and post- treatment facilities	
Digestate management	Residual products from the plants are bulky and are not easier to manage, handle, store, transport and use	Residual products from the plants are easier to manage, handle, store, transport and use	The digestates from the high solids AD plants have better utility values and therefore are marketable than digestates from wet AD plants
Retention time	The overall retention time was longer in the wet AD plants than in high solids AD plants	The overall retention time was shorter in the high solids plants AD than in the wet AD plants	The overall retention time for all the AD plants varied from 10 to 100 days
Parasitic energy consumption	The parasitic energy consumed by the wet AD plant was not significantly different from the one consumed by high solids plants	Relatively high parasitic energy demand of high solids plant was due to extensive pre- and post treatment processes	Parasitic energy is the produced energy consumed to operate the plants. The parasitic energy consumed by high solids AD plants was similar to the one consumed by wet AD plants
Biogas yield	Wet AD plants had greater biogas yields per tonne of waste processed than high solids plants. Specific biogas and methane yields (per tonne TS or VS) for wet AD plants were also higher than that of high solids plants	The average biogas yield for the high solids plants was 78 m^3^ per tonne of waste	The annual biogas yied which was dependent on plant capacity varied from 1.6 to 15.5 million m^3^
Economy performance	Specific capital cost per tonne of waste processed and specific capital cost per m^3^ of biogas produced by the wet AD plants were lower when compared to that of the high solids plants The average specific capital cost per tonne of waste processed by wet AD plants was £22 per tonne of waste input	The average specific capital cost per tonne of waste processed by high solid plants was £35 per tonne of waste input	Total capital cost of facilities for wet and high solids AD processes ranged from £54 to £42.7 million, The specific capital cost for the AD processes varied from £19 to £42 per tonne of waste input

Meanwhile, most of the challenges limiting the optimal performance of LS-AD could be overcome through the application of HS-AD with associated environmental, social and economic benefits [15–17]. One of the environmntal benefits of HS-AD, besides being environmental friendly method of waste management and a source of green energy; is that it helps in the conservation of water as low amount of water is needed for the process. Conservation of resources, such as water is essential for human survival and sustainablity. Regarding the social benefits, HS-AD plants/facilities are not complex when compared to LS-AD, and could also be made mobile since little amount of water is involved in the process. and as such, people living in remote areas will not have problem accessing social benefits including job creation, effective waste management, public health improvement and accessibility to electricity and heating energy, among others from the application of the LS-AD. The economic benefit of HS-AD when compared to LS-AD is the potential cost reduction from various aspects of the digestion process which include; reactor smaller footprint due to closely packed digester content, lower heat and mixing energy consumption, easy handling of feedstock and digestate transport; high biogas production rate as a consequent of high organic loading rate, as well as reduced equipment maintainance due to limited number of complex equipment such as pump and mixers [18].

Optimal performance of HS-AD has been limited by various factors including drastic increase in accumulated volatile fatty acids, requirement of high innocula concentration, extended solid residence time, and inefficient mixing due to heterogeneous nature of solid waste feedstock [19–21]. In order to overcome these barriers to optimal performance of HS-AD, extensive research work has been carried out with various measures suggested. However, there is a dissertation dearth on garnering the possible measures for easy domestic and industrial applications of HS-AD. The purpose of this review is therefore to assemble the various recommended measures to overcome HS-AD limitations for efficient industrial application of HS-AD. Furthermore, the review will propose a design protocol for effective application of HS-AD.

## Basics of HS-AD process

**Fig. 1:**
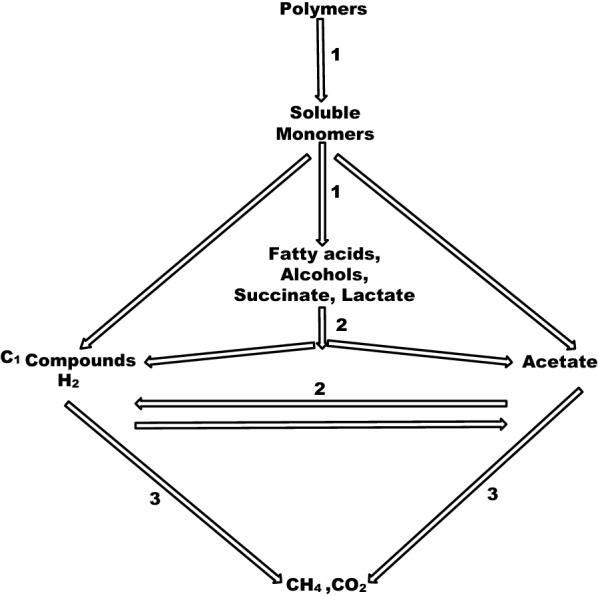
The AD Process through different degradation steps and trophic groups of microorganisms. 1 – Hydrolysis and Acidogenesis 2—Acetogenesis 3—Methanogenesis. Adapted from Schnürer and Jarvis [22]

Anaerobic digestion (AD) process involves the degradation of organic fraction of waste streams by microbial consortium (bacteria, archaea and most likely fungi) [22] for production of energy rich compounds, as well as, nutrient rich organic fertilizer. This AD process encompasses four sequential steps including hydrolysis, acidogenesis, acetogenesis and methanogenesis, respectively, with hydrolysis and acidogenesis being the main limiting steps of this process [23–28]. During the first two steps, carbohydrates, proteins and fats are converted into sugars, aminoacids, glycerol and long chain fatty acids and thereafter converted into alcohols, hydrogen and carbondioxide. Throughout the third step, the acids and alcohols are converted by acetogens to hydrogen, carbondioxide and acetate which are finally degraded by methanogens to biogas (mainly methane and carbondioxide) as shown in Fig. 1 [22, 25, 29].

There are various microorganisms which by synergetic interactions convert large organic macromolecules into smaller organic compounds and finally into biogas [22]. However, the dominant microorganisms in HS-AD processes may vary from those in LS-AD; there are changes in microbial morphology as the total solid content changes [30–32]. Fermentative or hydrolytic bacteria produce extracellular enzymes including amylases, proteases, and lipases which break down the insoluble organic compounds into soluble compounds. Acidogenic bacteria are a mixture of facultative and obligatory bacteria, which are important in creating an anaerobic conditions during the fermentation process since the facultative anaerobes have the potential of using up the oxygen that might have been introduced into the process through the feedstock. Bacteria response to an increase in hydrogen concentration in the medium results in the accumulation of electrons by compounds such as lactate, ethanol, propionate, butyrate, and higher volatile fatty acids [33]. Acetogenic bacteria including *Syntrophomonas* and *Syntrophobacter* convert products from acidogenesis into acetates and hydrogen and carbon dioxide which may be used by methanogenic bacteria [34]. Acetogenesis requires efficient and continuous removal of hydrogen formed from the fermentation process, as the process can only be favoured thermodynamically at low partial pressure of hydrogen. Therefore, a symbiosis between acetogenic bacteria that produce hydrogen and autotrophic methane bacteria that consume hydrogen is required. Methanogenic bacteria, consisting of the hydrogenotrophic and acetotrophic, are responsible for methane production from two pathways including cleavage of acetate to CH_4_ and CO_2_ and reduction of CO_2_ with hydrogen gas, which are known as acetoclastic methanogenesis and hydrogenotrophic methanogenesis, respectively. The preferred pathway depends on the type of feedstock, available microbial consortium and the process conditions during the digestion process [35].

HS-AD processes involves the treatment of wastes as received (usually wastes streams with TS content ≥ 20%); the process is more robust and flexible in acceptance of feedstock compared to the LS-AD processes [36]. However, the rheological properties of sludge with high solids content are important in determining the sludge performance in digesters [37]. Sludge with high solids content is a pseudoplastic fluid that shows a yield stress and viscosity (η) of 20 Pa s unlike sludge with TS content ≤ 10% which behaves like a Newtonian fluid with pumpable and mixable slurry in the digester [37, 38].

## HS-AD process challenges and control measures

The challenges associated with HS-AD process can be related to the environmental and operational factors. HS-AD process may be inhibited by change in temperature, pH and ammonia concentration but the inhibition is not so different from that obtainable from LS-AD process. So, the major challenge is related to feedstock composition, handling, pumping and mixing the high solid waste streams [39].

HS-AD is affected by environmemtal factors such as temperature, pH and composition of the feedstock. Generally, the optimum range values for the various factors are 35 and 55 °C for mesophilic and thermophilic temperature zones, respectively [40]; pH of 7.0–8.5[41]; nutrient composition in terms of C:N ratio of 23 to 30:1 [42]; retention period of 45–55 days at 25–30 °C, 20 days at 35–37 °C and 6–10 days at 55 °C [43]; slurry concentration of 8–10% [44]. For a stable and productive anaerobic digestion process, these factors should be maintained at optimal values.

A deviation from the optimum temperature range often lead to reduction in volatile fatty acid production rate, substrate decomposition rate, and metabolic rate of the microorganism [45, 46]. Variation in optimum temperature around ± 5 °C can result in reduction in biogas yield to 24% [47]; especially with thermophilic processes [48]. This challenge can be minimised using a two-phase anaerobic digestion process in which the hydrolysis/acidogenesis stage is operated under thermophilic conditions and the methanogenic stage is operated under mesophilic conditions [48, 49]. However, one stage system is used industrially because of cost implications. More so, part of biogas produced can be used in heating the reactor and maintaining constant temperature within the system; this is a good option for HS-AD since less energy is needed for heating due to low water content.

### Feedstock composition

HS-AD process is flexible in acceptance of feedstocks but inadequate preparation of feedstock before feeding can lead to blockage in the inlet zone of the reactor as well as blockage in the gas pipes thereby causing a significant reduction in biogas yield and a great effect on the overall digestion process [50]. Pretreatment of feedstock such as magnetic separation, comminution in a rotating drum or shredder, screening, pulping, gravity separation or pasteurization needs to be done prior to digestion [39]. Also, presence of inhibitors (e.g. antibiotics, disinfectants, solvents, herbicides, salts, and heavy metals) in the feedstock can reduce the microbial activity at high concentrations thereby affecting the process degradation rate. Sometimes, the high solids feedstock could be a dewatered sewage sludge from the wastewater treatment plant which might contain polyelectrolytes (substances added to water to enhance floc adhesion) [51]. Polyelectrolytes reduce the hydrolysis rate and affect methane production during the digestion process [52]. The sewage sludge may also contain flocs of microbial aggregates that could form a shield around the microbial aggregates preventing them from being degraded [53, 54].

Little amount of water in the sludge, which is a typical characteristic of HS-AD, during anaerobic digestion process could affect the rheological characteristics of the digester medium and cause acid accumulation leading to the failure of the process [55]. During HS-AD process, high solid content of the feedstock are converted to high volume of metabolites including volatile fatty acids (VFA), long chain fatty acids, (LCFA), hydrogen (H_2_), hydrogen sulfide (H_2_S) and ammonia (NH_3_), with much of the metabolites left to accumulate while only little quantity is further converted. Volatile fatty acids (VFA) are unavoidable intermediates formed during anaerobic digestion [8, 56] and unregulated high solid digestion process may result in accumulation of VFAs, including acetate, propionate and butyrate. Unlike acetate and butyrate, propionate cannot be easily converted, and as a consequent, it forms a larger proportion of the accumulated VFA [13, 14, 17]. The VFA production during anaerobic digestion may reduce the pH level to a point that may result in process inhibition since some inhibitors like hydrogen sulfide (H_2_S) becomes toxic when the medium pH is below 9 [57]. This condition could lead to process failure, with consequent failure in methane production, if it is not properly managed [18, 58, 59]. Usually, the rate of VFA production and consumption should be balanced for stable digestion process, and to prevent process failure, the medium alkalinity is buffered. However, care must be taken when buffering the medium alkalinity since accumulation of inorganic cations and anions from nutrients, or pH buffer added to the sludge medium may cause the substances to act as inhibitors to the activities of the anaerobic microorganisms [60].

Besides VFA accumulation, anaerobic digestion of high solids sludge can cause ammonia build-up that inhibits the activities of methanogenic bacteria [61]. Microbial degradation of organic feedstock is impeded by the diffusion of accumulated ammonium ion or free ammonia nitrogen into the microbial cells resulting in proton (H +) imbalance or potassium (K +) deficiency. The buildup of total ammonia nitrogen (TAN) in digester medium may cause accumulation of volatile fatty acids and consequent failure of the digester [62–68]. It has also been observed that high solid AD system sometimes allows the development of specific fermenting species of Clostridium [18] as a way of adapting to the high TS content. Hence, the HS-AD process is either operated in batch or continuous mode initiated with a start-up in each case for optimum performance [69]. Increasing the TS in the reactor gradually is a good process for enhancing microbial adaptation; this can be achieved by starting with a low TS and increase gradually to a high TS by steadily adding a high solid feedstock. Rivard, Himmel [70] reported a loading rate of 9.5 gVS/ld at 32%TS when the TS in reactor was gradually increased from 5 to 32% while treating municipal solid wastes under mesophilic condition.

Additional measures to minimize the effect of these challenges on the digestion process include co-digestion with other substrates which may help to provide the necessary buffering capacity or essential nutrients necessary for the growth of the anaerobic microorganisms [42]. More so, addition of activated carbon or zeolite as a support structure for inhibitors will reduce the process inhibitions thereby enhancing biogas yield and production rates [71].

### Mixing in HS-AD processes

Generally, gentle mixing promotes effective distribution of microorganisms and substrates throughout the digester ensuring close uniform heat transfer, as well as, release of gaseous products, which can also be enhanced by intermittent mixing of the digester contents [72, 73]. HS-AD process hardly takes place in a homogenous phase due to high viscosity, rheology of the solid wastes and the absence of a mixer [74]. Conventional mixer does not enhance homogeneity and may result in inadequate contact between the organisms and the substrates. Hence, poor mixing during HS-AD due to reduced water content can cause difficulties in heat and mass transfer resulting in process instability [75]. Good homogenization of the fresh feedstock with the digested waste in the reactor will enhance adequate inoculation thereby preventing overloading and particle sedimentation. Hence, mixing in HS-AD processes is enhanced through biogas injestion, inoculum and digestate recirculation thereby improving the decomposition of the solid wastes by active microbial consortium.

## Industrial applications of HS-AD processes

HS-AD Technologies are anaerobic digestion technologies developed out of the necessity to treat wastes with high solid content (≥ 20%) effectively; the theoretical concepts involve handling anaerobic digestion processes at high solid loadings, as well as reduceing nutrient loss and water content in digestate. Details on each of these technologies coupled with their achievement in overcoming major challenges with HS-AD processes and current challenges associated with them are shown in Table 2. Typical HS-AD industrial technologies are Bekon, Biocel, Bioferm, Dranco, Kompogas, Valorga and Strabag among others [76–81]. Strategies adopted by each of these technologies in designing their reactors to overcome major challenge with mixing in HS-AD processes are evaluated in this section.

**Table 2:** Comparison among different single stage HS-AD technologies

S/N	Technology/Location	Process Mode	Capacity ton/day	Feedstock/ %TS dry matter	Temp-rature (ºC)	SRT day	OLR kgVS /m^3^d	Methane yield m^3^/kg VS removed	HS-AD challenges addressed	HS-AD current challenges	Refs.
1	BEKON system/Germany	Batch	20.6–109.6	Agricultural waste/N/A	37–55	28–35	N/A	0.17–0.37	(i) It reduces the system complexity and machinery maintenance requirement (ii) The problem of poor start up performance is reduced through the recirculation of leachate	The challenges of incomplete mixing and accumulation of VFAs due to high solids content of the feedstock still remains The batch mode of the process, as well as, the recirculation of the percolate could allow the buildup of inhibitory compounds in the system	[89]
2	BIOCEL/Netherlands	Batch	95.9	OFMSW/30–40	35–40	15–21	N/A	N/A	The problem of mass transfer limitation due to high solids content of feedtock is improved through the recirculation of percolate	The problems of VFA build up which can affect the stability of the process still exists in the batch mode digester	[90–93]
3	BIOFerm/Germany	Batch	21.9	OFMSW/25	37	28	N/A	0.21–0.35	In addition to enhancing the startup performance, the technology improves the cost efficiency of the process through effective use of heat to maintain percolation temperature and energy input	The introduction of heat exchanger for effective heat usage could add to the cost of the procurement	[94, 95]
4	DRANCO/Belgium	Continuous	27.4 -191.8	OFMSW/10–32	50–55	20	10–15	0.21–0.30	The process does not require the introduction of mixers in the digester, thereby, eliminates the complexity of the system configuration. Mixing is done outside the digester through the addition of percolate to the fresh feedstock	The issue of incomplete mixing due to the inherent poor mass transfer limitation of the high solids feedstok still present. This makes the system structure to be complex and increases process and maintenance cost	[90–92, 96–100]
5	KOMPOGAS/Switzerlamd	Continuous	N/A	OFMSW/30	55	20	4.3	0.39–0.58	The poor mixing issue associated with the digestion process is addressed through the usage of impellers in the horizontal digester. Stratification problem is also reduced during the process	The introduction of impellers increases maintenance cost, thereby, reduces the overall cost effectiveness of the process	[92, 98, 101]
6	VALORGA/France	Continuous	54.8- 958.9	OFMSW/36–60	37–55	20–33	10–15	0.21–0.30	The system does not require percolate recirculation. Proper mixing of contents is done using injectors supplying biogas under high pressure every 15 min into the digester	Biogas injectors introduced into the system are often associated with clogging which require maintenance	[90, 92, 98, 102–104]

**Fig. 2:**
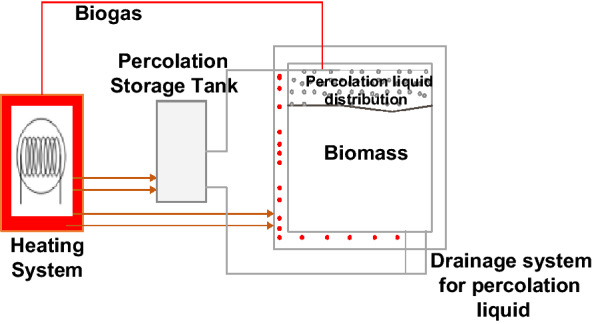
One Phase Batch Reactor Design with integrated heating and percolation liquid systems. BEKON System ( Adapted from BEKON [81])

The BEKON system adopts a batch reactor design that can process bulk waste streams with high solid content at both mesophilic and thermophilic conditions; this system ensures continuous biogas production by running several reactors simultaneously [82]. Heating and constant temperature were maintained within the system through heated floor and walls as shown in Fig. 2. The process is non-mixed; liquid from the digestate is drained and recirculated back into the reactor to provide moisture for the microoorganisms [82]. BEKON system is robust and as such distinguished by its reliability in operation and low investment and operating costs. However, there are still some challenges with incomplete mixing resulting in sedimentation and possible VFAs accumulation that needs to be addressed for better-quality application.

BIOCEL system adopts a similar reactor design to that of BEKON system for treating source separated organic fraction of municipal solid wastes (OFMSW) of TS 30 – 40% as shown in Table 2. The process is non-mixed but percolation liquid is collected and recirculated back continuously into the reactor. The system was initially set up to reduce cost through material handling simplification, high loading rates and elimination of the need for mixing equipment. The major challenge currently associated with this design is the high accumulation of volatile fatty acids during the hydrolysis/acidogenesis stage as indicated in Table 2.

BIOFerm system is also batch type digestion system similar to that of BEKON; it is well suited for feedstock with solids content of 25 – 35%. It operates in the mesophilic temperature range and percolation recirculation system is designed to recover residual heat from combined heat and power [78]; it is an effective heat usage thereby reducing energy input. Liquid percolation also help in moistening the microorganisms for improved performance in the reactor system. However, there is still challenge with possible VFA accumulation and cost of procurement of heat exchangers as indicated in Table 2.

**Fig. 3:**
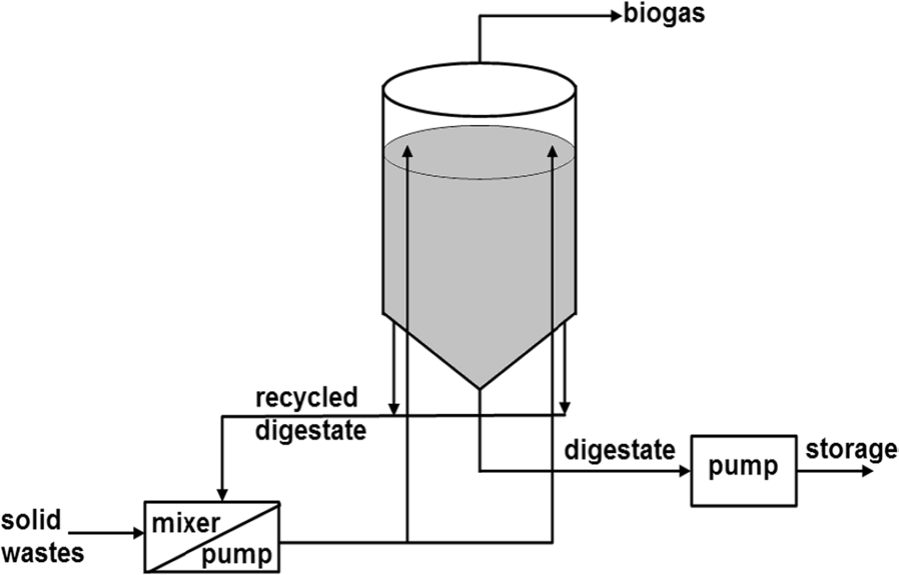
Reactor designs for adequate mixing of solid wastes; DRANCO System. (Adapted from Baere [81])

DRANCO system is a continuous HS-AD process with total solid content of 15% to 40% in the reactor [81]; this system has three main characteristics including its vertical design, high-solids concentration, as well as the absence of mixer in the reactor as shown in Fig. 3. Mixing is enhanced in the system through digestate recirculation [83]; one part of fresh wastes mixed with six parts of the digestate. This is an improved design as it eliminates the mass transfer limitation as recorded in the batch systems mentioned previously.

KOMPOGAS system, the process is similar to that of DRANCO system except that the reactor is design horizontally; it is a horizontal plug flow reactor. This design incorporates low speed impellers for homogenisation and degassing [39, 83, 84]; the digesting mass is moved by vortex flow in a horizontal position. Feeding fresh substrates into the reactor continuously pushes the digestate slowly through the reactor to the outlet. This design improves the mass and heat transfer in the system thereby eliminates the challenge of particle sedimentation and allows homogeneity. Some notable plants implementing Kompogs technology include the ones in Braunschweig-Watenbuttel (Germany) with 20,000 tons per year capacity; Tyrol (Austria) with capacity of 10,000 tons per year capacity; as well as, the plants in Switzerland which are Bachenbulach, Otelfingen and Volketswil with capacities of 13, 577, 13, 814 and 9,377 tons per year, respectively.

**Fig. 4:**
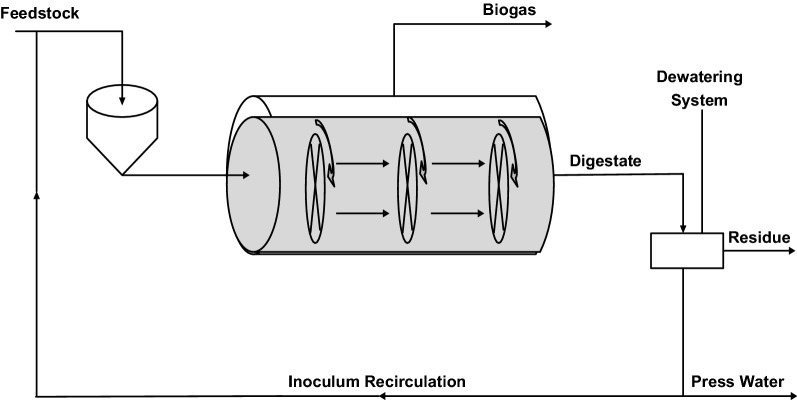
Horizontal plug flow bioreactor design with inoculum recirculation; STRABAG System (Adapted from STRABAG [85])

STRABAG System is a continuous reactor designed to treat dry wastes of 15 to 50% total solid content [85]. The design principle is also based on horizontal plug flow as in the case of KOMPOGAS System except that it is equipped with agitators which are arranged transversely to the flow direction as shown in Fig. 4; this enhances mixing thereby preventing uncontrolled sedimentation. The overlapping arrangement of the agitators ensures mixing of the substrate, optimum release of the gas and reliable transportation of digestate to the outlet. The agitator drives operate intermittently and thus with very low energy consumption [85]. However, there is need for further development of this technology to increase the methane content in biogas thereby making the process cost effective.

**Fig. 5:**
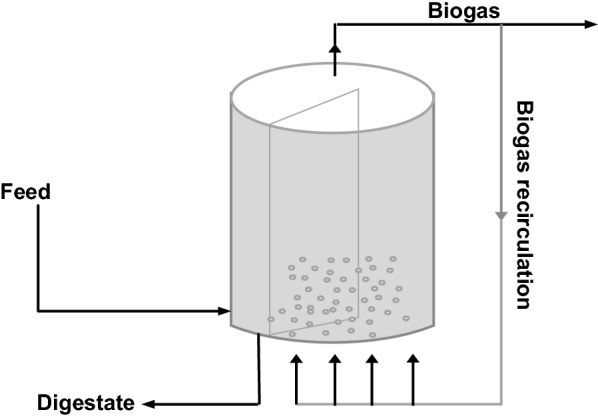
Vertical plug flow bioreactor design with biogas recirculation; VALORGA System (Adapted from Vandevivere, De Baere [83])

VALORGA system is a single stage continuous HS-AD process (Fig. 5); it is a cylindrical vertical reactor but with a circular horizontal plug flow system. Mixing occurs through biogas injection (at every 15 min) [39] with high pressure at the bottom of the reactor. Mixing is well enhanced with this system but the major challenge could be with clogging of injectors. The benefits of these various design systems in enhancing HS-AD industrially are highlighted in Table 2 together with the current challenges that need improvement.

## Proposed design for HS-AD industrial plants

**Fig. 6:**
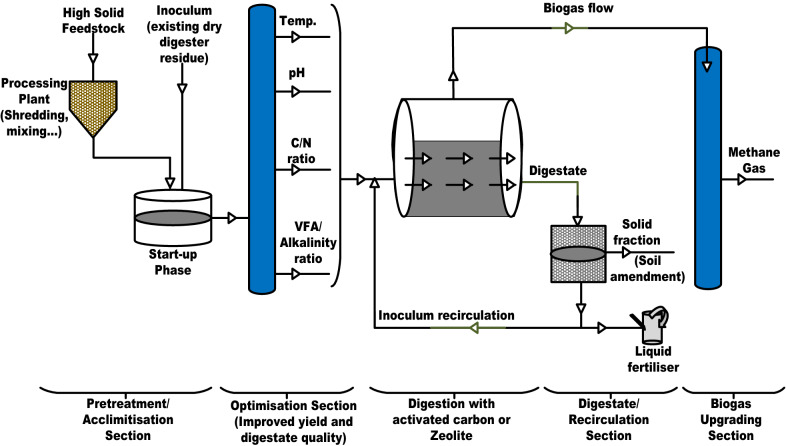
Proposed Design for Limiting Inhibition in HS-AD Plants

High solids anaerobic digesters must be designed to overcome possible limitations that may be encountered during the anaerobic digestion process to ensure optimal performance. Because of the feedstock heterogeneity, as well as, operational and environmental parameters that affect the anaerobic digestion process, the importance of pretreatment section cannot be understated as the key to the success of the HS-AD process. The pretreatment section will ensure that the feedstock for the HS-AD plants has suitable parameters including biodegradability, composition, fluid dynamics, among others, since the microbial anaerobic digestion process of the feedstock depends on these parameters. Some of the industrial processes explained in the previous section adopt the start-up phase for enhancing industrial adaptation. Inoculum recirculation is also adopted but addition of activated carbon or zeolite as shown in Fig. 6 will help in the absorption of possible inhibitors thereby improving the stability of the process. The biogas upgrading facilities is necessary to remove possible contaminants in the biogas produced so that challenges during biogas utilization will be minimal. Water moisture can be removed by cooling in heat exchanger; water absorption using glycol or water adsorption using adsorbers such as silica gel, activated carbon, molecular sieves, aluminium oxide or magnesium oxide. Activated carbon or molecular sieves can be used to remove siloxanes, particulate and gaseous contaminants such as halogenated hydrocarbons, ammonia, and organic silicon compounds. Hydrogen sulphide can be removed using different iron species (iron chloride, iron hydroxide, or oxide) while carbon dioxide can be removed using methods such as membrane separation, pressure swing adsorption, polyethylene glycol scrubbing or water scrubbing.

## Economic implications of HS-AD process

HS-AD process has several socio-economic and environmental benefits including provision of renewable biogas fuel for transportation, electricity and heat energy; production of nutrient-rich fertilizer for agricultural purposes; provision of waste management services that reduce greenhouse gas emission from degradation of organic material, as well as, generating revenues from the sales of biogas (electricity and heat energy), among others. However, the benefits of the HS-AD process may be outweighed and affected by the capital and operating costs for the process, if adequate planning of the process is not done. It has been determined that capital costs range between £400 and £800/ton/year of installed capacity [86] while operating cost depends on the anaerobic digestion plant either wet or dry; and was estimated to vary from $18 to 100/ton of feedstock handled by the plant [87]. Examples of capital cost include property purchase, lift station pumps, plant installation, digester and mixing tanks, generators, and flow meters, among others; while operating cost includes cost of feedstock, logistics for feedstock and products, cost for maintenance and repairs, among others.

Among the dry anaerobic digestion technologies, the most applied technologies that have feedstock TS range of 30–40% are Dranco, Kompogas and Valorga [88]. The capital and operating costs incurred by any company involved in HS-AD process will depend on the type of technologies adopted by the company. For example, two companies implementing Dranco technology in Safzburg (Austria) and Brecht (Belgium) having capacity of 20,000 and 20,049 tons per year, respectively, and operating at temperature of 55 °C; will have to set up substantial amount for the digester capacity and maintenance, as well as, for the heat energy required to maintain the process at thermophilic temperature. Additional, the BEKON technology does not require pumps and mixers for operation, even the bulk waste does not require any pretreatment. So, the machines and operating costs are less expensive compared to the wet fermentation process. In view of this, a detailed economic analysis on these HS-AD technologies should be carried out in order to evaluate the cost of adopting these technologies; cost estimates can be obtained from stimulation models. There is also need to further develop these technologies to increase the methane content of the biogas, this will potentially drive down the cost of HS-AD process to be economical.

## Conclusion and perspectives

In this review, the theoretical and industrial concept of the HS-AD process was addressed putting into considerations the available technologies. Thereafter, the challenges associated with the HS-AD process were highlighted and available technologies addressing such challenges currently in the industries were appraised. More so, design concept to overcome current limitations to optimal performance of the HS-AD process was proposed; this is vital for a wider acceptance and application of the HS-AD technology in the industries. However, there is a need to further investigate and direct research work towards establishing optimum values for various HS-AD operational parameters such as total solid content of the feedstock, organic loading rate, solid retention time, VFA/Alkalinity ratio, liquid, biogas or digestate recirculation depending on the type of reactor design adopted. Industries can control the process parameters automatically based on the optimum values obtained from research in order to avoid process failure and maximize profit. This is necessary for optimum performance of the HS-AD process and economic viability. Also, the metabolic pathways for digestion of carbohydrates, proteins and lipids in HS-AD processes should be known in order to maximize the digestion process.

## Data Availability

Not applicable.

## Abbreviations

AD: Anaerobic digestion
HS-AD: High solid anaerobic digestion
LS-AD: Low solid anaerobic digestion
TAN: Total ammonia nitrogen
C/N: Carbon to nitrogen ratio
LCFA: Long chain fatty acids
OLR: Organic loading rate
STR: Solid retention time
TS: Total solids
VFA: Volatile fatty acids
VS: Volatile solids
